# Analysis of factors that promote the participation of patients with chronic diseases in shared decision making on medication: a cross-sectional survey in Hubei Province, China

**DOI:** 10.1186/s12889-023-17099-0

**Published:** 2023-12-06

**Authors:** Qijun Hu, Zhanchun Feng, Qiao Zong, Jia Wang, Zehao Zheng, Da Feng

**Affiliations:** 1 School of Medicine and Health Management, Tongji Medical College, Huazhong University of Science and Technology, Wuhan, Hubei China; 2 Science and Education Department, Traditional Chinese and Western Medicine Hospital of Wuhan, Tongji Medical College, Huazhong University of Science and Technology, Wuhan, 430014 Hubei China; 3 School of Pharmacy, Tongji Medical College, Huazhong University of Science and Technology, Wuhan, Hubei China

**Keywords:** Shared decision making, Chronic disease, Random forest

## Abstract

**Background:**

Shared decision making (SDM) improves the health status of patients with chronic diseases, especially in the condition of poly-medicated patients. This study aims to find the factors associated with participation of patients with chronic diseases in SDM on medication.

**Methods:**

A total of 1,196 patients with chronic diseases were selected in Hubei Province of China using cluster sampling methods. The random forest method was applied to rank the importance of independent variables by Mean Decrease Gini and out-of- bag (OOB) curve. Multivariate logistic regression was used to explore the independent variables’ effect direction and relative hazard.

**Results:**

In this study, 5.18% of patients used patient-directed decision making (PDM, a decision-making model led by patients), 37.79% of patients used SDM (a collaborative decision-making model by patients and doctors), and 57.02% of patients used doctor-directed decision making (DDM, or paternalistic decision making, a decision-making model led by doctors). The random forest analysis demonstrated that the top 5 important factors were age, education, exercise, disease course, and medication knowledge. The OOB curve showed that the error rate reached minimum when top 5 variables in importance ranking composed an optimal variable combination. In multivariate logistic regression, we chose SDM as a reference group, and identified medication knowledge (OR = 2.737, 95%CI = 1.524 ~ 4.916) as the influencing factor between PDM and SDM. Meanwhile, the influencing factors between DDM and SDM were age (OR = 0.636, 95%CI = 0.439 ~ 0.921), education (OR = 1.536, 95%CI = 1.122 ~ 2.103), exercise (OR = 1.443, 95%CI = 1.109 ~ 1.877), disease course (OR = 0.750, 95%CI = 0.584 ~ 0.964), and medication knowledge (OR = 1.446, 95%CI = 1.120 ~ 1.867).

**Conclusion:**

Most Chinese patients with chronic diseases used DDM during their medication decision-making, and some patients used PDM and SDM. The participation in SDM should be taken seriously among elderly patients with lower education levels. The SDM promotion should focus on transformation of patients’ traditional perception and enhance their medication knowledge.

## Background

Chronic diseases are a group of diseases with insidious onset, long duration, and persistent symptoms [1]. The US Institute of Health Metrics and Evaluation (IHME) reported that 72% of global mortality was associated with chronic diseases in 2020 [2]. Effective treatments for chronic diseases are essential worldwide. Currently, medication remains the most commonly used treatment to alleviate chronic disease symptoms. However, many elderly patients with chronic diseases are prescribed multiple medications leading to complex medication regimens. This complexity could result in medication safety issues, such as polypharmacy and non-compliance with medication [3, 4], ultimately resulting in an increased incidence of adverse drug reactions (ADRs) [5].

Our previous study has discovered a negative correlation between polypharmacy behaviors and shared decision making (SDM) among chronic disease patients in community [6]. This study was conducted to further explore the factors influencing chronic disease patients’ participation in SDM on medication based on a similar cohort. SDM refers to a collaborative process in which patients and their doctors discuss the pros and cons of various medical regimens, consider patients’ values and preferences, and finally make medical decision together [7]. Furthermore, participants in SDM may also involve multiple medical staff and patients’ social networks [8]. However, some evidences have showed that most patients reported lower levels of SDM and retained doctor-led views on decision-making, especially engrained in the elderly [9, 10]. Although low levels of SDM among elderly patients with chronic diseases have garnered attention from researchers, several studies focused on implementing programs to enhance patients’ experience in the SDM process, rather than figuring out what factors stimulate patients to participate in this process [11–13]. Considering that patients' participation is the foundation of SDM, we believe that the first step is to identify the factors that promote patients to participate in SDM, and then take corresponding measures to improve the participation in SDM among elderly patients with chronic diseases.

China has the largest elderly population in the world, facing the critical challenge of chronic diseases [14]. In 2009, the Chinese government launched the National Essential Public Health Service, implementing packages of interventions for the management of chronic diseases, including health education, improving medication compliances, and developing healthy habits [15]. Current studies indicated that the chronic disease population in China showed improvements in health knowledge, medication compliances, and health habits [16]. However, few studies have focused on whether these improvements influence the participation of patients with chronic diseases in SDM [13]. It is also unclear which of these are the key contributing factors to the SDM process. In this context, we decided to select Hubei province of China as a sample area to analyze the impact of above factors on SDM on medication. Based on the importance of the factors, we have made some recommendations to promote the participation of chronic disease patients in SDM on medication, thereby improving the health status of patients.

## Methods

### Study design and data collection

From April to June 2021, we conducted a face-to-face questionnaire survey of community patients by using a cluster sampling method in Hubei Province, China. In this study, we focused on the factors influencing decision-making on medication in patients with chronic diseases, including knowledge, health behaviors, and so on. Given that health services in rural areas are poorer than in urban areas, urban and rural areas were divided to carry out the sampling process [17]. Our sampling process was consistent with two previous studies based on similar cohorts [3, 6]. Firstly, we categorized Hubei Province into urban and rural areas, and then randomly selected 2 cities in each of category. Wuhan and Yichang were selected as sample urban areas, while Zhijiang and Qianjiang were selected as sample rural areas. Secondly, we followed the same simple randomization process to choose 3 districts in each of the four selected cities, resulting in a total of 12 districts for our survey. In each of the selected districts, we recruited patients with hypertension or diabetes from primary health care providers. Inclusion criteria included: (1) adults aged 18 years and above. (2) ability to express themselves clearly. (3) taking medicines for a long time (more than 3 months) due to chronic diseases. A total of 1,260 invitations were sent to patients with chronic diseases through primary care in all sample districts, resulting in 1205 patients agreeing to participate, a response rate of 95.6%. All of participants completed the questionnaires, 9 of which were excluded due to incomplete information. Effective responsive rate was 99.3%. All participants in this survey were required to complete an informed consent form or provide verbal consent to participate in the survey.

### Demographic data

Demographic and disease-related data, including age, gender, education, domicile, residence status, job type, medical insurance, disease course, exercise, and drink, were collected through our questionnaire. In addition, according to Age-Based Grouping Criteria of World Health Organization and the average life expectancy in China at the end of 2019 [18, 19], we categorized participants into three age groups: adults (< 65 years), young-old (65–75 years), and oldest-old (≥ 75 years). We categorized patients’ residence status as living alone or not living alone according to whether they live with family.

### Measurement of variables

We used the Control Preference Scale-Post (CPSpost) [20, 21], a modified version of Control Preference Scale (CPS), to assess the type of decision-making models of patients with chronic diseases, that is, the actual control of doctors and patients over medication decisions, which was perceived by patients. Previous studies indicated that CPSpost is a valid and reliable scale to measure the participation of patients in medical decision-making [21, 22]. A total of five entries are included as follows: (1) I made my medication decision alone; (2) I made my medication decision alone considering what my doctor said; (3) I shared the medication decision with my doctor; (4) My doctor decided considering my preferences; (5) My doctor made the medication decision. (1) and (2) were categorized as PDM (a process in which the patient is the initiative role in the decision-making), (3) was categorized as SDM, (4) and (5) were categorized as DDM (a process in which the doctor is the initiative role in the decision-making) [23]. We asked patients to answer based on two experiences as follows: (1) communication with doctors when prescribed medications for the first time; (2) communication with doctors during medication adjustments over the past three months.

Medication knowledge was evaluated through a questionnaire adapted from the study by McPherson et al., a total of seven entries and codes were shown in Table 1. According to McPherson’s classification method, we used the median score as the threshold to separate medication knowledge into high and low score groups of medication knowledge [24].

**Table 1:** Medication knowledge questionnaire

Entry	Score
**Could you tell me the name of medications you are taking?**	
Don't know	0
Knows the name of the medication	1
**Could you tell me why you take this medication?**	
Don’t know	0
Can describe the indications, such as lower hypoglycemia	1
Can describe exactly how the medication works	2
**Do you know how to take your medication?**	
Don’t know	0
Knows how to take, such as brewing or swallowing	1
**Do you know when to take your medication?**	
Don’t know	0
Knows when to take, such as fasting	1
**Could you tell me what side effects your medication may cause?**	
Don’t know	0
Can describe side effects of medications	1
**Could you tell me what to do if side effects occur?**	
Don’t know	0
Can describe specific measures, such as discontinue	1
**Do you know what to do if you miss a dose of your medication?**	
Don’t know or says "double the dose"	0
Never misses a dose or says "carry on as usual" or "ask doctor or pharmacist for advice"	1

Medication compliance was examined using the 4-item Morisky medication adherence scale (MMAS-4) [25], which was widely used to measure the medication compliance of patients with chronic diseases and has presented favorable among Chinese patients [26]. The detailed entries of the scale were as follows: (1) Do you ever forget to take your medicine? (2) Are you careless at times about taking your medicine? (3) When you feel better do you sometimes stop taking your medicine? (4) Sometimes if you feel worse when you take the medicine, do you stop taking it? For each item, we assigned the answer of “Yes” as 0 point, and assigned the answer of “No” as 1 point. We divided medication compliance into high and low score groups on medication compliance according to the distribution of total score.

Depression symptom was measured by Short Version of Center for Epidemiological Studies Depression Scale (CESD10), which met strict clinical requirement [27].

### Statistical analysis

We used Pearson’s χ2 test to conduct descriptive analysis of demographic characteristics and other variables in different decision-making model groups.

Random forest (RF) is a machine learning method for noise immunity, prevention of over­fitting, and independence from co-linearity, which showed a preference for important predictor variables by Gini coefficient, and applied to any significance tests and variable selection [28]. We used the RF method for two main reasons. Firstly, when compared with other variable selection models, RF is a machine learning method that covers the impact of each predictor variable individually as well as in multivariate interactions with other predictor variables and thus work towards the global optimality of the variable selection [29]. Secondly, RF provides relative importance among variables, which is of great value in targeting interventions. In this study, we used Mean Decrease Gini (MDG) and out-of-bag (OOB) curve to select variables, which was proposed by Hong Han et al. [29]. Gini coefficient is an indicator reflecting to the degree of inconsistency in the sample categories on the node, the lower of Gini coefficient, the better results of classification [30]. MDG refers to the total decrease of Gini from splitting on the variable averaged over all trees, which is used to indicate the importance of the predictor variable to the response variable [29]. OOB error rate is used to estimate the prediction error of current model by using the set of remaining samples which are not included in current tree [29]. We firstly referred to MDG to rank the importance of predictor variables, then selected the most appropriate number of variables to be included in the multivariate logistic regression model according to OOB curve. The multivariate logistic regression with *P* value, OR, and 95% CIs was used for analyzing the predictor variables’ effect direction and relative hazard.

Moreover, due to the proportion of decision-making models is unbalanced (62 PDMs, 452 SDMs and 741 DDMs). The imbalance of categories will affect the classification effect of RF —— the classification result tends to favor the majority category. Therefore, we used the synthetic minority sample oversampling method (SMOTE) to balance the data. The SMOTE method is a data preprocessing technique applied to imbalance problems proposed by Chawla et al. [31], which uses the K- nearest neighbors and linear interpolation to add minority class samples to balance the class distribution [32]. In R’s smotefamily package, we set the K parameter to 3 and dup_size to 6, which means the minority class will generate 6 times as many new samples based on 3 original samples from random nearest neighbors. Finally, we obtained 372 new PDMs, a total of 432 PDMs included in RF.

R (version 4.0.3, R Project for Statistical Computing) and SPSS (version 24.0) were used for all statistical analyses in this study.

## Results

Among 1196 participants, 57.94% participants were female, 48.33% resided in the urban area, and 62.63% were manual workers. The average age of participants was 68.55 years old (ranging from 26 to 92 years). The majority of patients used DDM (57.02%), while some patients used SDM (37.79%), and a smaller percentage opted for PDM (5.18%). Other detailed information was shown in Table 2.

**Table 2:** Characteristics of the study population

Variables	PDM N (%)		SDM N (%)		DDM N (%)		total	χ2	*P*
Age									
<65	14	(22.58)	133	(29.42)	148	(21.70)	295	10.783	0.029
65–75	33	(53.23)	236	(52.21)	372	(54.55)	641		
≥ 75	15	(24.19)	83	(18.36)	162	(23.75)	260		
Gender									
Male	33	(53.23)	196	(43.36)	274	(40.18)	503	4.480	0.106
Female	29	(46.77)	256	(56.63)	408	(59.82)	693		
Education									
Primary school or below	24	(38.71)	172	(38.05)	347	(50.88)	543	24.065	< 0.001
Middle school	27	(43.55)	149	(32.96)	185	(27.13)	361		
High school or above	11	(17.74)	131	(28.98)	150	(21.99)	292		
Domicile									
Urban area	35	(56.45)	221	(48.89)	322	(47.21)	578	2.035	0.361
Rural area	27	(43.55)	231	(51.11)	360	(52.79)	618		
Residence status									
Live alone	7	(11.29)	39	(8.63)	78	(11.44)	124	2.367	0.306
Not live alone	55	(88.71)	413	(91.37)	604	(88.56)	1072		
Job									
Manual worker	44	(70.97)	255	(56.42)	450	(65.98)	749	12.573	0.002
Brain worker	18	(29.03)	197	(43.58)	232	(34.02)	447		
Medical insurance									
Employee health insurance	31	(50.00)	198	(43.81)	258	(37.83)	487	6.354	0.042
Resident health insurance	31	(50.00)	254	(56.19)	424	(62.17)	709		
Disease course									
≤ 10 years	31	(50.00)	249	(55.09)	321	(47.07)	601	6.998	0.030
> 10 years	31	(50.00)	203	(44.91)	361	(52.93)	595		
Drink									
Never	41	(66.13)	364	(80.53)	542	(79.47)	947	11.066	0.026
Occasionally	11	(17.74)	63	(13.94)	93	(13.64)	167		
Always	10	(16.13)	25	(5.53)	47	(6.89)	82		
Exercise									
Never	7	(11.29)	52	(11.50)	93	(13.64)	152	12.728	0.013
Occasionally	18	(29.03)	157	(34.73)	286	(41.94)	461		
Always	37	(59.68)	243	(53.76)	303	(44.43)	583		
Medication knowledge									
Lower	41	(66.13)	194	(42.92)	372	(54.55)	607	20.884	< 0.001
Higher	21	(33.87)	258	(57.08)	310	(45.45)	589		
Compliance									
No	42	(67.74)	180	(39.82)	260	(38.12)	482	20.792	< 0.001
Yes	20	(32.26)	272	(60.18)	422	(61.88)	714		
Depression									
No	40	(64.52)	357	(78.98)	475	(69.65)	842	14.324	0.001
Yes	22	(35.48)	95	(21.02)	207	(30.35)	324		

### Importance ranking of the independent variables

**Fig. 1:**
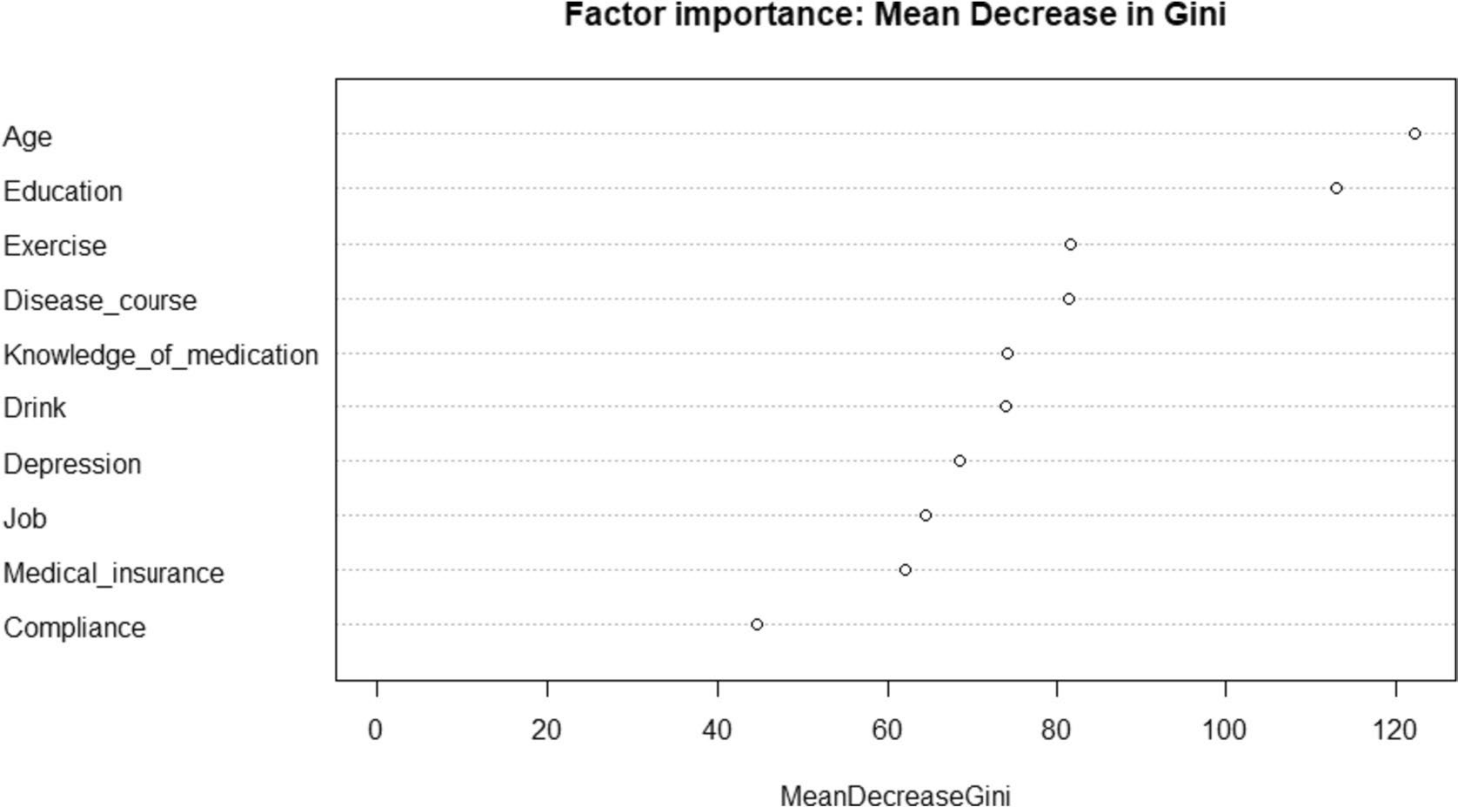
The importance of factors influencing decision-making on medication

Ten independent variables with a *p*-value less than 0.05 in univariate analysis were included in RF analysis, with ntree as 500. Figure 1 showed the visualization results of the importance ranking of 10 variables. According to the results of MDG, the top 5 important variables were age, education, exercise, disease course, and medication knowledge.

**Fig. 2:**
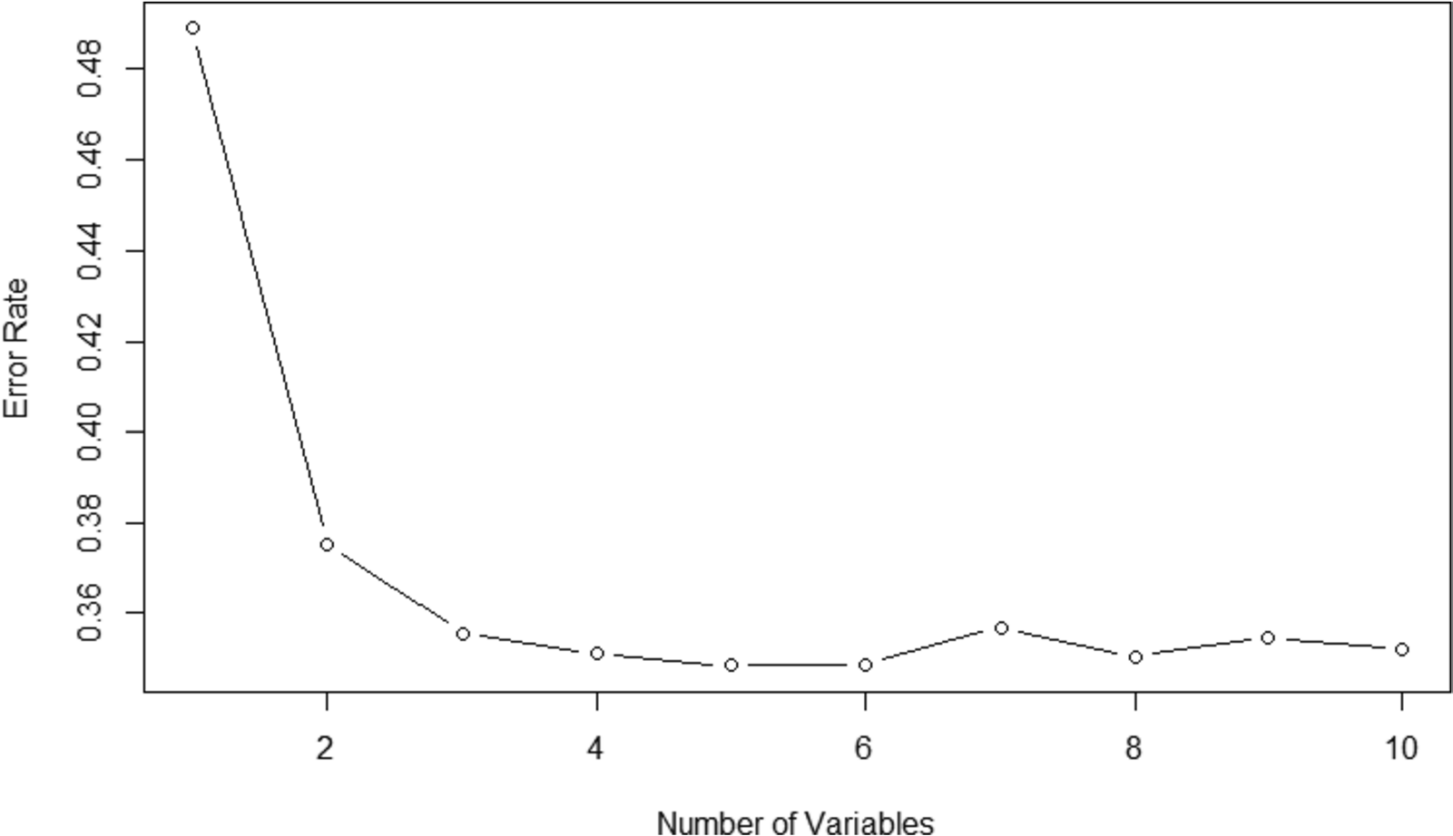
Relationship of OOB error rate with number of variables

Figure 2 showed that OBB error rate was lowest when model contained 5 variables. The top 5 variables in order of importance ranking were: age, education, exercise, disease course, and medication knowledge.

According to the importance ranking and OOB curve of RF, 5 independent variables (age, education, exercise, disease course, and medication knowledge) were included in multivariate logistic regression.

### Influencing factors of decision-making model

We chose the SDM group as a reference group in multivariate logistic regression analysis since SDM was considered to be a hallmark of patient-centered care and more advocated during the clinical encounter compared with other two decision-making models.

Table 3 showed the significance of factors influencing chronic patients’ participation in medication decision-making. The patients with lower medication knowledge (OR = 2.737, *P* < 0.05) were more likely to use PDM than SDM.

**Table 3:** Multivariate logistic regression of factors associated with decision-making on medication

Variables	Types	PDM			DDM		
		(ref: SDM)			(ref: SDM)		
		β	OR(95%CI)	P	β	OR(95%CI)	*P*
Age	≥ 75 (ref)						
	<65	-0.528	0.590(0.264 ~ 1.319)	0.199	-0.453	0.636(0.439 ~ 0.921)	0.016
	65–75	-0.326	0.722(0.368 ~ 1.417)	0.343	-0.174	0.841(0.610 ~ 1.158)	0.288
Education	High school or above (ref)						
	Primary school	0.194	1.214(0.558 ~ 2.641)	0.625	0.429	1.536(1.122 ~ 2.103)	0.007
	or below						
	Middle school	0.662	1.939(0.914 ~ 4.111)	0.084	0.06	1.062(0.766 ~ 1.472)	0.718
Disease course	> 10 years(ref)						
	0–10 years	-0.226	0.798(0.461 ~ 1.382)	0.421	-0.288	0.750(0.584 ~ 0.964)	0.025
Exercise	Always (ref)						
	Never	-0.373	0.689(0.285 ~ 1.664)	0.407	0.196	1.216(0.822 ~ 1.801)	0.327
	Occasionally	-0.332	0.718(0.391 ~ 1.316)	0.284	0.366	1.443(1.109 ~ 1.877)	0.006
Medication knowledge	Higher (ref)						
	Lower	1.007	2.737(1.524 ~ 4.916)	0.001	0.369	1.446(1.120 ~ 1.867)	0.005

When compared with DDM, the patients under 65 years (OR = 0.636, *P* < 0.005) and disease course under 10 years (OR = 0.750, *P* < 0.005) were more likely to participate in the SDM during the medication decision-making process. By contrast, patients with infrequent exercise (OR = 1.443, *P* < 0.05), lower educational levels (OR=1.536, P<0.05) and poor medication knowledge (OR = 1.446, *P* < 0.05), were more likely to use DDM.

## Discussion

In this study, 57.02% of patients used DDM during their decision-making process, which was lower than similar research conducted in other Asian countries, such as the United Arab Emirates and Japan [33, 34]. It may be related to the success of the National Essential Public Health Service launched by the Chinese government in the last decade, which enhanced the family doctor signing rate and residents’ health literacy [35, 36], thus providing an external environment and internal motivation for patients’ participation in SDM. The percentages of PDM reported in other studies were around 15%, higher than the 5.18% in this study [34, 37–39]. It might because of a higher proportion of elderly patients in our study, their views on medical decision-making were more conservative and passive [9]. As the patient-led decision-making model, PDM required knowledge and deliberation of patients [40]. Otherwise, it was difficult to make informed decisions. Therefore, when compared with PDM, SDM, as a collaborative process between patients and doctors, was more advocated during the clinical encounter. In this study, only 37.80% of patients with chronic diseases used SDM during their decision-making on medication. This was a lower level compared with some western countries [38, 41]. A cross-­sectional study conducted in Sweden showed that 38.6% of patients with chronic diseases experienced the SDM process during their clinical encounters [41]. Another study conducted in Sweden reported a higher percentage of SDM reached 47% [38].The reason might be that western countries launched SDM earlier in clinical practice, and many tools, programs, and regulations have been provided to promote patients’ participation in SDM [13, 42]. However, previous studies showed that patients had a positive attitude toward SDM [38], which suggested opportunities to promote SDM in patients with chronic diseases to improved patients’ health status. Therefore, we focused on the factors associated with patients’ participation in SDM in this study.

According to the results of RF, the most influencing factor identified was the age of patients. Logistic regression showed that the patients under 65 years old were more likely to use SDM compared with the patients over 75 years old. One of the explanations for passive participation of elderly patients might be the poor ability to seek and understand information [11]. On the one hand, seeking disease-related information would promote the patients to participate in SDM since patients would be well prepared for their treatments [43, 44]. However, elderly patients had limited ability to seek disease-related information, especially through the Internet, which acted as a barrier to SDM [45]. On the other hand, many elderly patients preferred to leave the decision-making to their doctors since a lot of treatment information caused “information overload” to them [46]. Consequently, they tended to make faster decisions by their doctors when compared with the younger [11]. Moreover, the stereotype of the “patient” rooted in elderly patients might also be a key reason for the passive participation [9]: most elderly patients believed that a “good” patient should be compliant and passive and questioning doctors frequently would be labeled as “difficult patients” and led to a worse care. Thus, this misconception of the doctor-patient relationship prevented elderly patients from participating in SDM. Therefore, the enhancement of information literacy and transformation of traditional perceptions should be taken seriously in the SDM process for elderly patients with chronic diseases.

Our results showed that the patient’s educational level was the second most relevant variable. Patients with higher educational levels were more likely to use SDM when compared with DDM, which was consistent with the results of other studies [47, 48]. A possible explanation would be that well-educated patients usually have higher health literacy (the ability to access and utilize medical information). The well-educated patients knew what information they wanted to access from their doctors during their clinical encounters, and understood the information provided by their doctors during the decision-making process [47]. Thus, healthcare professionals should use appropriate methods of communication when serving patients with different education levels to encourage less educated patients to participate in SDM.

Our results further showed that the exercise frequency was the third most relevant variable. The results of logistic regression suggested that chronic disease patients who always exercise were more likely to use SDM when compared with DDM. China has promoted integrating active health into chronic disease management, which refers to transforming patients with chronic diseases from a passive role in their treatment to an active role [49]. Regular exercise is a common active health behavior [50]. Patients with chronic diseases who always exercise tended to prioritize their own power and responsibility in the treatment process [51, 52]. Thus, they often desired more information about the disease during their clinical encounters [51]. Moreover, exercise may also promote patients' participation in decision-making by influencing patients’ self-efficacy [52]. Previous studies indicated that achievement of short-term exercise goals would promote patients' confidence in controlling diseases, resulting in more participation in further medical decision-making [53]. Furthermore, patients who always exercise were more likely to maintain better cognitive and physical functions, which is essential for participation in decision-making.

This study has also suggested that chronic disease patients with a course of under 10 years were more likely to use SDM when compared to DDM. Patients with a disease course over 10 years were more likely to have a deteriorating condition, mainly manifested by cognition and sensory, which reduced patients’ confidence to participate in SDM, as well as doctor’s patience [54, 55]. As a result, doctors might be more reluctant to initiate SDM with patients with poor cognitive. Moreover, as the disease worsens, patients’ treatment options might be more limited [55]. This was also one of the reasons for the passive participation of patients with a long-term course of disease.

The results suggested that patients’ medication knowledge influenced decision-making models. Logistic regression showed that the patients with lower levels of medication knowledge were more likely to use DDM than SDM, which was consistent with the findings from previous studies [13, 56, 57]. This could be attributed to lower medical knowledge that undermined patients’ confidence during clinical encounter [58]. They perhaps believed that their opinions were worthless to the doctor and treatment process. Additionally, complex medical jargon and names of medications were viewed as barriers to decision-making, especially for those patients with lower medication knowledge [59]. Moreover, our study also found that patients with lower medication knowledge were more likely to use PDM as compared with SDM. This might be due to patients who lack medication knowledge were more likely to be influenced by negative information, such as adverse reactions and side effects, thus making their own decisions to stop or replace medications [60, 61]. However, given that our results were based on correlation analysis, another possible explanation was that participating in SDM improved patients’ medication knowledge. Indeed, medication education through SDM has been launched among patients with chronic diseases in some countries [62], while previous studies reported that there was limited effectiveness of education in SDM process due to pressurized healthcare environment and inadequate capacities of medical staff [13, 63]. Therefore, better preparation for decision-making, such as providing patient decision aids (PDAs), was more advocated [13]. Although PDAs have attracted the attention of researchers since the 1990s [64]. Few PDAs were designed for chronic disease patients in mainland China at present, esecially for their medication decision-making [65]. Therefore, developing PDAs for medication decision-making in the context of Chinese cultural background and healthcare system would be a meaningful research direction in the future.

### Strengths and limitations

In this study, we used a combination of RF and logistic regression model to find out the key factors associated with the participation in medication decision-making of patients with chronic diseases. This was a special feature compared with other SDM related research. The data were collected from 12 districts of 4 cities in the Hubei Province, China, which are representative of all patients with chronic disease in entire Hubei Province. However, there are still several limitations in this study. Firstly, this study only focused on the impact factors of participation in decision-making from the patients’ perspective, not paying much attention to the factors from healthcare providers. Secondly, this study recruited patients voluntarily. The patients who weren’t willing to participate were not surveyed, which may generate some data bias. Finally, logistic regression did not reported significance in some PDM results, which may be attributed to the small sample size of patients using PDM (only 62 cases, 5.18% of the total sample). It is important to expand the PDM sample in future studies.

## Conclusion

According to the findings in this study, the key factors associated with SDM were age, education, exercise, disease course, and medication knowledge. Based on the results, several corresponding interventions could be taken to improve patients' participation in medication decision-making. Firstly, doctors should pay more attention to elderly patients with lower education levels, and encourage them to participate in SDM. Secondly, health education should focus on transforming patients’ traditional perceptions and behaviors to enhance their awareness of participation in SDM. Finally, development and application of PDAs to improve patients' medication knowledge and promote them to participate in SDM will be an important topic in further research and clinical practice.

## Data Availability

The datasets used and/or analyzed during the current study are available from the corresponding author on reasonable request.

## Abbreviations

SDM: Shared decision making
IHME: Institute of Health Metrics and Evaluation
PDAs: Patient decision aids
PDM: Patient-directed decision making
DDM: Doctor-directed decision making
RF: Random forest
MDG: Mean Decrease Gini
OOB: Out-of-bag
